# The Local Pathology of Neuralgia

**Published:** 1844-11

**Authors:** 


*The Local Pathology of Neuralgia* has been explained by Dr. Black upon anatomical principles. He very justly observes, that the nerves, which are usually the seat of neuralgic pains, are those which take their exit from the interior of the body through canals in bone or unyielding tendinous structure. He adds to this, the anatomical fact, that each nervous twig is accompanied by a branch of an artery and a vein. It may easily, therefore, be conceived that those nerves, which are contained in rigid canals, must be subjected to injurious pressure whenever their accompanying vessels are unusually distended with blood. Upon this pressure, according to Dr. Wallis, depends the neuralgic paroxysm. The explanation is, I think, borne out by the consideration both of the exciting cause and the effects of treatment.

Dr. Ranking in *Ibid.*

